# Spatial Heterogeneity of Government Regulation, Spatial Distance and Enterprise Carbon Information Disclosure: An Analysis Based on the Heavy Pollution Industry in China

**DOI:** 10.3390/ijerph16234777

**Published:** 2019-11-28

**Authors:** Quanqi Liu, Li Li

**Affiliations:** 1 School of International Business, Guangdong University of Finance & Economics, Guangzhou 510320, China; 2 School of Economics and Management, Harbin Institute of Technology, Shenzhen 518055, China; liszsz@hit.edu.cn

**Keywords:** government regulation, spatial heterogeneity, spatial distance, carbon information disclosure

## Abstract

Under the background of the construction of ecological civilization in China, since China has a vast amount of territory and large distances between cities, the intensity of environmental supervision in different regions may affect the enthusiasm of enterprises to disclose carbon information. Based on the listed companies of the Chinese heavy pollution industry from 2009 to 2014, using the content analysis method, the paper researches the influence of the spatial heterogeneity regarding government regulation and spatial distance on enterprise carbon information disclosure and puts forward some suggestions. The empirical results show that carbon information disclosure is significantly positively related with the spatial heterogeneity of government regulation. The spatial heterogeneity of government regulation is significantly positively associated to non-financial carbon information disclosure. The relationship between government supervision and financial carbon information disclosure is not significant. Spatial distance and carbon information disclosure are significantly positively related. There is also a significant positive correlation between spatial distance and financial carbon information disclosure. Further study finds that in public enterprises, the spatial heterogeneity of government regulation will promote carbon information disclosure, and the influence of spatial heterogeneity of government regulation on non-financial and financial carbon information disclosure both are significantly positively related. In non-public enterprises, spatial distance and carbon information disclosure are significantly positively related, and spatial distance and non-financial carbon information disclosure are significantly positively related as well.

## 1. Introduction

On 25 September 2015, China announced that the CO_2_ emissions per unit of gross domestic product (GDP) will decline 60%–65% from 2005 to 2030. In 2017, China launched the national carbon emissions trading system, covering key industries such as iron and steel, electric power, chemical, etc. [1]. Low-carbon development has become the consensus of Chinese people, and will also be the trend of enterprise development, and the implementation of the low-carbon development mode requires effective regulatory means. Environmental information disclosure is considered one of the important models of environmental regulation [2]. Now, developed countries such as the USA, Canada, and Australia have forced enterprises to disclose carbon information [3,4,5]. However, Chinese enterprises have shown little enthusiasm for disclosing carbon information. The extremely low response rate of Chinese enterprises to the Carbon Disclosure Program (CDP) is a good example. Only a few Chinese listed companies disclose “environmental information” in their corporate social responsibility reports, and most of them include only qualitative disclosure, which indicates that the initiative of enterprises is relatively low. The main reasons are as follows.

(1) The legal system of carbon information disclosure is not perfect, and the content and items of carbon information disclosure are not specified. Enterprises are under less legal pressure, and there is no uniform standard to follow. Although the “Law of Environmental Protection” implemented by the Ministry of environmental protection on 1 January 2015 and the “Environmental Information Disclosure Measures (Trial Implementation)” implemented on 1 May 2008 have made provisions for information disclosure, they are relatively general requirements. However, they have no specific provisions on carbon information, so they do not induce specific pressure on enterprises. 

(2) There are differences in the enforcement of policies and regulations among regions, industries, and enterprises. First, there are differences in the implementation of laws and regulations at the national level among local governments. Under the decentralization of Chinese power, the environmental regulation is used by local governments as a game tool to obtain resources, which leads to the differences in environmental regulation among different regions, resulting in a general phenomenon of incomplete implementation of environmental regulations [6]. The quality of carbon information disclosure is related to the intensity of government regulation [7]. The different regulatory pressure among regions is an important reason for the difference of carbon information disclosure level of enterprises [8]. 

Second, different industries have different requirements for carbon information disclosure. Due to the particularity of the heavy pollution industry, the government pays more attention to carbon emission reduction in the heavy pollution industry. Compared with other industries, the heavy pollution industry has received more attention from society, and its carbon information disclosure is also a hot topic of media coverage. 

Third, enterprises are also different regarding implementing the regulations of the state and local governments. There is always a certain distance between enterprises and government regulators. In a vast country such as China, such spatial distance is hard to ignore. Geographical location is the main factor affecting the level of environmental information disclosure [9]. These differences affect the enthusiasm of enterprises to disclose carbon information.

Within the existing literature, many have studied the factors affecting carbon information disclosure, which have been mainly related to the corporate characteristics [10], media pressure, and other factors [11,12], but few studies have been done on the factors affecting carbon information disclosure from the perspective of space. Based on the manufacturing listed companies from 2009 to 2011, Yao et al. analyzed the influencing factors of environmental information disclosure from the perspective of game theory, and considered the influence of geographical and institutional factors [9]. However, whether the research results are applicable to other industries remains to be investigated. The government should formulate differentiated environmental regulation standards according to the actual characteristics of various industries [13]. After 2012, China’s determination to tackle environmental pollution has been strengthened. Therefore, it is more realistic to increase the data after 2012 for research. Based on the listed companies of Chinese heavy pollution industries from 2009 to 2014, this paper will analyze the factors affecting the carbon information disclosure of enterprises from the perspective of space, enrich the research literature in this field, and provide information reference for the government’s environmental regulatory decision-making.

## 2. Theoretical Analysis and Research Hypothesis

### 2.1. Spatial Heterogeneity of Government Regulation and Carbon Information Disclosure

The government’s way of supervising enterprises is mainly to use environmental regulations to regulate the activities of enterprises. Environmental regulation has become an important tool for the government to manage the economy and society. Environmental regulation is a collection of all the rules and regulations on environmental protection. Research on the impact of environmental regulation is mainly focused on energy saving and emission reduction efficiency [14], enterprise transformation [15], productivity [16,17], competitiveness [18], enterprise performance [19], and environmental information disclosure [9]. However, the impact on carbon information disclosure is rare. Li et al. found that environmental regulation and carbon information disclosure are significantly negatively correlated [20]. Tang and Demeritt found that firms disclose their emissions in response to regulatory compulsion [21]. Yuan and Wang studied the impact of carbon emission systems on carbon information disclosure, and found that a perfect carbon emission system is positively related to carbon information disclosure, and the promotion effect of heavy pollution industry is more significant [22]. Li and Cheng also studied the legislative response to carbon information disclosure [23]. The power of making environmental regulations lies in the central government, and the local government is the specific implementation subject. Local governments have certain flexibility in implementing environmental regulation, which leads to differences in the implementation effects. Local government is not only the executor of environmental regulation, but also the actual supervisor of related work. The spatial heterogeneity of government regulation refers to the difference within the environmental regulation effect caused by different local governments’ different efforts in monitoring the implementation of environmental regulation. When Zhang studied the phenomenon of the “incomplete enforcement” of environmental regulation in China, he found that there was a significant complementary strategic interaction between regions. That is to say, the intensity of environmental regulation in competitive regions would be reduced accordingly, showing the contagiousness of the incomplete enforcement of environmental regulation [9]. The competition among local governments will affect the effective regulation of environment [24]. The enforcement of local government environmental regulation will also affect the dynamic evolution of the central and local governments in the process of implementing environmental regulation strategies [25]. Reducing the cost of environmental regulation and improving the benefits of environmental regulation will help to enhance the enforcement of local government environmental regulation [26,27]. 

The theory of information asymmetry holds that the party who has more information is in an advantageous position in economic activities. The carbon information disclosure market features information asymmetry, enterprises are in an advantageous position, and enterprises have the possibility of selectively disclosing carbon information. Under the condition of industry classification, the government should focus on formulating stricter environmental regulation policies for pollution industries [28]. Environmental regulation will affect the transformation mode of different enterprises and promote the transformation of enterprises [15]. Some scholars have found that institutional factors are the main factors affecting the content and extent of environmental information disclosure [9]. Jia et al. analyzed the strategic changes and evolutionary trends of government supervision departments and enterprises and found that environmental information disclosure supervision will affect enterprise cost, particularly government supervision cost [29]. Voluntary information disclosure theory holds that enterprises may voluntarily disclose environmental information to avoid regulatory risks. Government environmental regulation is an important factor affecting voluntary environmental information disclosure [30]. The degree of environmental regulation in different regions is different, and the degree of impact on enterprise environmental performance information disclosure is different [31]. The government’s formulation and implementation of environmental supervision policies can play a positive role in environmental information disclosure [32]. Therefore, the following assumptions are proposed:

**Hypothesis** **1:** *Government regulation can promote carbon information disclosure, and the impact of government regulation on non-financial carbon information disclosure is more significant than financial carbon information disclosure.*

Considering the different nature of listed companies, the level of carbon information disclosure may be affected by the intensity of government regulation [33]. On the one hand, public-owned enterprises are easily concerned by the public, and will disclose more carbon information to meet the public’s attention needs; on the other hand, public-owned enterprises are often the benchmark in the industry, and their behavior has a certain guiding role for other enterprises in the industry. The government will strengthen supervision, so that public-owned enterprises play a model leading role. At present, the government pays special attention to environmental protection-related work, among which, strengthening the enterprise to disclose carbon information is very important work. Therefore, public-owned enterprises will actively cooperate with government regulation and disclose more carbon information, while the interaction between non-public-owned enterprises and the government is not obvious.

**Hypothesis** **2a:** *In public-owned enterprises, the stricter the government supervision, the higher the level of carbon information disclosure. The level of non-financial carbon information disclosure and financial carbon information disclosure is positively correlated with government supervision, respectively.*

**Hypothesis** **2b:** *In non-public-owned enterprises, the relationship between government regulation and carbon information disclosure level is not significant, and the relationship between the level of non-financial and financial carbon information disclosure and government regulation is not significant.*

### 2.2. Spatial Distance and Carbon Information Disclosure

Neoclassical economics theory holds that information is the main factor affecting investment decision-making behavior. There is information asymmetry between enterprise managers and investors. The geographical location of enterprises is an important aspect to reflect the degree of information asymmetry [34]. The information asymmetry theory holds that the information asymmetry between enterprises and external investors will lead to an irrational behavior of market participants. The geographic location of enterprises can affect the degree of information asymmetry between enterprises and investors [35]. The degree of information asymmetry of remote enterprises is higher than that of big cities, and the geographical location aggravates the degree of information asymmetry [36]. The higher the degree of information asymmetry, the more obvious the influence of geographical distance [37]. Geographical location can be measured by spatial distance. Spatial distance refers to the distance between the enterprise and the government. If the enterprise is a central enterprise, it refers to the distance between the enterprise and the central government. If the enterprise is a provincial or municipal enterprise, it means that the distance between the enterprise and the government is at the corresponding level. China’s territory is large, and the spatial distance between enterprises and government management departments is also quite different. Geographical location is negatively correlated with the selective disclosure of environmental information [9]. The spatial distance between the office space of listed companies and institutional investors will have a negative impact on the investment behavior of institutional investors [38]. Yao and Liang believe that the geographical location plays a decisive role in the process of the impact of environmental information disclosure on the synchronization of stock prices [39]. 

Voluntary information disclosure theory holds that enterprises may voluntarily disclose environmental information to avoid regulatory risks. Therefore, enterprises with long spatial distances may adopt the strategy of voluntary disclosure of more environmental information to avoid regulatory risks. Reputation is an important factor affecting the social information of enterprises. According to reputation theory, reputation is a scarce and inimitable precious resource, which can win competitive advantage for enterprises in many similar distributors [40]. From the perspective of long-term interests, all parties to the transaction will restrain their short-term opportunistic behaviors, so that trading partners can form a “good view” (good reputation) of them, so as to achieve long-term cooperation benefits [41]. Based on different theoretical foundations, we propose the following two opposite assumptions:

**Hypothesis** **3a:** *According to the voluntary information disclosure theory and reputation theory, spatial distance is positively correlated with carbon information disclosure.*

**Hypothesis** **3b:** *According to the information asymmetry theory, spatial distance is negatively correlated with carbon information disclosure.*

Spatial distance may affect the carbon information disclosure of listed companies of different natures. The corporate image of public-owned enterprises is relatively stable, and the influence of spatial distance is not obvious. However, the non-public-owned enterprises in remote areas are eager to get public recognition, and they will get a good image by disclosing more carbon information. Therefore, we propose the assumptions as follows:

**Hypothesis** **4a:** *In public-owned enterprises, the relationship between spatial distance and carbon information disclosure is not significant, and the relationship between non-financial and financial carbon information disclosure and spatial distance is not significant, respectively.*

**Hypothesis** **4b:** *In non-public-owned enterprises, spatial distance has a significant positive correlation with carbon information disclosure. At the same time, spatial distance has a more significant impact on non-financial carbon information disclosure than financial carbon information disclosure.*

## 3. Research Design

### 3.1. Sample Selection and Data Sources

The sample includes the listed enterprises in China’s heavy pollution industry in 2009–2014. As the production process and products of enterprises in the heavy pollution industry are relatively special, they need to bear more corporate social responsibility and become a typical sample of carbon information disclosure. Excluding Special Treatment (ST) and Delisting early warning (*ST) enterprises, the number of enterprises is 128, and the total number of enterprises is 768. Carbon information disclosure data are obtained from Corporate Social Responsibility (CSR) or Sustainable Development (SD) reports published on the enterprises’ websites by hand. The rest of data are from the database of China Stock Market Accounting Research (CSMAR) or Research Set (RESSET).

### 3.2. Definition and Measurement of Variables

#### 3.2.1. Carbon Information Disclosure 

Based on the previous research, this paper uses the same approach as Li et al. [42]. It takes the content analysis method to analyze the reports and construct the carbon information disclosure index, uses CID to represent the variable of carbon information disclosure, and then subdivides them into non-financial carbon information disclosure (CIDNF) and financial carbon information disclosure (CIDF), and uses the content analysis method to analyze the carbon information of sample enterprises. According to the actual situation, each disclosure item is given a score between 0 and 3, finally, it is used to calculate the specific index. The specific equations are as follows:

(1) $${\mathrm{CID}}_{i}=\frac{\sum {\mathrm{CIDP}}_{i}}{\mathrm{MCID}}$$

(2) $${\mathrm{CIDF}}_{i}=\frac{\sum {\mathrm{CIDFP}}_{i}}{\mathrm{MCIDF}}$$

(3) $${\mathrm{CIDNF}}_{i}=\frac{\sum {\mathrm{CIDNFP}}_{i}}{\mathrm{MCIDNF}}$$

where CID_i_ represents the carbon information disclosure of enterprise i; ΣCIDP_i_ is the total score of enterprise i; and MCID refers to the sum of the highest score among all the disclosure items in the Equation (1). The symbols of Equations (2) and (3) express similar meanings.

#### 3.2.2. Government Regulation 

The government regulation variable (GOV) is expressed by the pollution information transparency index (PITI), which indicates the regulatory results of government administrative departments on environmental information disclosure. Under the guidance of the “Government Information Disclosure Regulations” of the State Council and the “Measures for Environmental Information Disclosure” of the Ministry of Environmental Protection, the PITI is an evaluation of the environmental information disclosure of 113 cities in China, which has increased to 120 cities since 2013, in cooperation with the Institute of Public and Environmental Affairs (IPE) and the United States Natural Resources Defense Council (NRDC). The full score of PITI is 100, of which more than 60% of PITI is set according to the requirements of laws and regulations, while the rest is set according to the actual needs of the public. The PITI evaluation results are aimed at improving the information disclosure and transparency of the Chinese government. They have the characteristics of high credibility and reflect the level of government supervision.

#### 3.2.3. Spatial Distance 

Referring to the practice of Yao et al., space (Space) represents the shortest distance between listed enterprises and the government [9]. Specific practices are as follows: if it is a central enterprise, take the distance between the enterprise’s registered location and Beijing; if it is a provincial enterprise, take the distance between the enterprise’s registered location and the provincial capital; and for other enterprises, take the distance between the regional government where the enterprise is registered. Baidu Map (Baidu Inc., Beijing, China) is used to calculate driving distance in kilometers.

### 3.3. Control Variables 

The control variables of this study include profitability (ROA), financial leverage (LEV), enterprise growth (Growth), enterprise scale (Size), the proportion of first shareholders (First), board scale (Bsize), book-to-market ratio (BM), β coefficient (Beta), market process (MI), and the year listed (Year).

### 3.4. Model Construction

After all the variables are selected, in order to test the relationship between the spatial heterogeneity of government regulation, spatial distance, and corporate carbon information disclosure, the following model is constructed according to the research hypothesis:

(4) $$\begin{array}{cc} CID_{i,t}=a_{0} & +a_{1}GOV_{i,t}+a_{2}Space_{i,t}+a_{3}ROA_{i,t}+a_{4}LEV_{i,t}+a_{5}Growth_{i,t}+a_{6}Size_{i,t}\\ & +a_{7}First_{i,t}+a_{8}Bsize_{i,t}+a_{9}BM_{i,t}+a_{10}Beta_{i,t}+a_{11}MI_{i,t}\\ & +a_{12}Year_{i,t}+\varepsilon_{i,t}. \end{array}$$

## 4. Empirical Results and Analysis

### 4.1. Descriptive Statistics and Correlation Analysis of Variables

#### 4.1.1. Descriptive Statistics 

Table 1 shows the descriptive statistical results of variables, which is obtained by using SPSS software (SPSS Inc., Chicago, IL, USA). The mean of CID is 0.305 and the standard deviation is 0.193, which indicates that the enterprise carbon information disclosure of China’s heavy pollution is generally low; the mean of the CIDNF is 0.379 and the standard deviation is 0.219; the mean of financial carbon information disclosure (CIDF) is 0.178 and the standard deviation is 0.208. From the mean of carbon information disclosure and its classification, we can see that the main carbon information disclosure of enterprises is non-financial carbon information disclosure, while the financial carbon information disclosure is less. It shows that the key to improving the quality of carbon information disclosure is to promote the level of financial carbon information disclosure on the premise of maintaining the level of non-financial carbon information disclosure. The minimum value of GOV is 8.300, the maximum value is 85.300, the average value is 47.841, and the standard deviation is 17.029, which indicates that the degree of government supervision in different regions is quite different; the mean of the spatial distance is 3.575, and the standard deviation is 1.874, which indicates that the difference of spatial distance is great. From Table 1, we can also see that the data of Size, MI, and Year are quite different, but on the whole, most of the data are relatively stable.

#### 4.1.2. Correlation Analysis 

Table 2 shows the Pearson correlation coefficient between variables. The correlation coefficient between government regulation (GOV) and carbon information disclosure (CID) is 0.001, but it is not significant. The correlation coefficient between space distance and carbon information disclosure (CID) is 0.099, which is significant at the 1% level, indicating that the farther the space distance between enterprises and government, the higher the level of carbon information disclosure.

### 4.2. Multiple Regression Results

We use Equation (4) to analyze the relationship between government regulation, spatial distance, and carbon information disclosure. The regression results are as follows.

#### 4.2.1. Overall Sample Analysis 

From Table 3, we can see that the regression coefficient of government regulation (GOV) and enterprise carbon information disclosure (CID) is 0.094, which is significantly positive related at the 5% level (the T value is 2.114), indicating that strengthening government supervision will promote the quality of carbon information disclosure. The regression coefficient of GOV and CIDNF is 0.091, and they have a significant positive relationship at the 5% level (T value is 2.009), showing that the stricter the government supervision, the higher the level of non-financial carbon information disclosure. The regression coefficient of GOV and CIDF is 0.072, but it is not significant. In a word, the stricter the government regulation, the better the quality of carbon information disclosure, and the influence of government regulation on non-financial carbon information disclosure is more significant than on financial carbon information disclosure. Therefore, research hypothesis H1 has been verified.

Table 3 shows that the regression coefficient of spatial distance (Space) and carbon information disclosure (CID) is 0.069, which is significantly positively correlated at the 5% level (T value is 1.976), indicating that the farther the spatial distance, the better quality of the enterprise carbon information disclosure, verifying research hypothesis H3a. In other words, H3b is not supported by empirical research. At the same time, the regression coefficient of spatial distance and non−financial carbon information disclosure (CIDNF) is 0.045, which is not significant; the regression coefficient of spatial distance and financial carbon information disclosure (CIDF) is 0.082, which is a significant positive correlation at the 5% level (T value is 2.264). The results show that the impact of spatial distance on financial carbon information disclosure is more significant than that of non−financial carbon information disclosure.

#### 4.2.2. Analysis of Public-Owned Enterprises Sample 

In Table 4, it can be seen that the regression coefficient of government regulation (GOV) and carbon information disclosure (CID) is 0.112, which is significantly positively correlated at the 5% level (T value is 2.070), indicating that strengthening government supervision will promote the enthusiasm of the carbon information disclosure of public-owned enterprises. The regression coefficient of GOV and CIDNF is 0.099, and they are significantly positively related at the 10% level (T value is 1.810), showing that the greater the intensity of government regulation, the higher the quality of non-financial carbon information disclosure. The regression coefficient of GOV and CIDF is 0.096, and there is a significantly positive relationship at the 10% level (T value is 1.744), indicating that the strengthening of government regulation can also improve the financial carbon information disclosure of public-owned enterprises. In short, strengthening government regulation can promote the quality of the carbon information disclosure of public-owned enterprises, and significantly improve the quality of the non-financial and financial carbon information disclosure of public-owned enterprises. The research hypothesis H2a has been verified.

In Table 4, we can see that the regression coefficient of spatial distance (Space) and carbon information disclosure (CID) is 0.022; the regression coefficient of Space and CIDNF is −0.010; the regression coefficient of Space and CIDF is 0.058, but both of them are not significant. The research hypothesis H4a is verified.

#### 4.2.3. Analysis of Non-Public-Owned Enterprises Sample 

The results in Table 5 show that the regression coefficient of government regulation (GOV) and carbon information disclosure (CID) is 0.042, the regression coefficient of GOV and CIDNF is 0.074, and the regression coefficient of GOV and CIDF is −0.018. However, the results are not significant. Research hypothesis H2b is verified.

It can be seen from Table 5 that the regression coefficient of spatial distance (Space) and carbon information disclosure (CID) is 0.191, which is significantly positively correlated at the 5% level (T value is 2.428), indicating that the farther the spatial distance, the higher the carbon information disclosure level of non-public-owned enterprises. The regression coefficient of Space and CIDNF is 0.202, and they are significantly positively related at the 5% level (T value is 2.525), indicating that the farther the spatial distance, the better the quality of non-financial carbon information disclosure of non-public enterprise. The regression coefficients of Space and CIDF is 0.110, but they are not significant. The results show that the impact of spatial distance on the non-financial carbon information disclosure of non-public-owned enterprises is more significant than the influence of spatial distance on financial carbon information disclosure. Thus, research hypothesis H4b has been verified.

### 4.3. Sensitivity Test

To check the stability of the results, this study recalculates the spatial distance between the listed enterprise and the government by referring to the method of Ivkovic and Weisbenner to replace the spatial distance (Space) [43], and the substitution variable is expressed by Spacer. Table 6 shows the regression results, and the test results indicate that the signs of regression coefficients have not changed, no matter whether it is for the overall sample, the public-owned enterprise sample, or the non-public-owned enterprise sample. There is not much difference between the test results and the previous empirical results, which shows that the previous research results are stable.

## 5. Conclusions 

In recent years, the government of China has attached great importance to environmental protection. Many enterprises have begun to implement the low-carbon development strategy and disclose the carbon information of enterprises. The intensity of government regulation and spatial distance have become an important factor affecting the carbon information disclosure of enterprises. This study takes the listed companies of the Chinese heavy pollution industry from 2009 to 2014 as a sample to test the impact of the spatial heterogeneity of government regulation and spatial distance on carbon information disclosure. The empirical results show that government regulation can promote carbon information disclosure. The results are consistent with the findings in previous studies in the literature [15,22,31,32,33]. After classifying the carbon information into non-financial and financial carbon information, we also found that the relationship between government regulation and non-financial carbon information disclosure is significantly positive; the relationship between government regulation and financial carbon information disclosure is not significant. Strengthening environmental regulation by the government will improve carbon information disclosure, and the performance of non-financial carbon information disclosure is more obvious. Spatial distance has a positive influence on carbon information disclosure. The results are consistent with the voluntary information disclosure theory and reputation theory. However, the relationship between spatial distance and non-financial carbon information disclosure is not significant. Spatial distance is positively related to financial carbon information disclosure, and the impact of spatial distance on financial carbon information disclosure is more significant.

Further, the listed enterprises are divided into public-owned enterprises and non-public-owned enterprises. It is found that on the one hand, for public-owned enterprises, strengthening government regulation will promote the quality of enterprise carbon information closure, and it will significantly improve the quality of non-financial and financial carbon information disclosure. However, the impact of spatial distance on the carbon information disclosure of public-owned enterprises is not significant, and the effect of it on non-financial and financial carbon information disclosure is also not significant. On the other hand, in non-public-owned enterprises, the relationship between government regulation and carbon information disclosure, as well as that between non-financial and financial carbon information disclosure are not significant, respectively. At the same time, the farther the spatial distance, the better the quality of non-financial carbon information disclosure, while the relationship between spatial distance and financial carbon information disclosure is not significant.

In a word, enterprise carbon information disclosure will be affected by government regulation and spatial distance. However, different types of carbon information are impacted differently, and the performance of this difference is not the same in different types of enterprises. 

## 6. Discussions

According to the results, we propose the following recommendations. First, the government departments should adopt the way of differentiation when formulating environmental regulations. Although the way of strengthening the supervision has promoted the carbon information disclosure of public-owned enterprises, it is not necessarily effective for non-public-owned enterprises. Second, the government and industry should explore the means to make the content of carbon information disclosure concrete and quantitative together, and enhance the operability and comparability of carbon information disclosure. Third, the government should strengthen the construction of the carbon information disclosure system. As the difference of spatial distance is objective, enterprises may voluntarily disclose carbon information to avoid regulatory risk. The construction of the carbon information disclosure system plays an important role in regulating the behavior of carbon information disclosure and minimizing the impact of spatial distance on carbon information disclosure. Fourth, the government should increase the number of inspections on the environment of the more distant enterprises. Since enterprises far away are often less supervised by the public, the regulatory departments should take the initiative to increase the amount of inspections. Finally, strengthening the moral education of specific supervisors is necessary so that the effect of system implementation can be guaranteed.

Based on the voluntary information disclosure theory and information asymmetry theory, this study expands the existing research literature from three aspects. First, based on the characteristics of heterogeneity, the research attempts to test the impact of government regulation on carbon information disclosure, financial carbon information disclosure, and non-financial carbon information disclosure, respectively; second, according to the actual spatial distance, it examines the influence of spatial distance on carbon information disclosure; third, this paper explores the effect of the spatial heterogeneity in relation to government regulation and space distance on the carbon information disclosure of enterprises of different natures, respectively. Therefore, this paper enriches the research literature in the field of carbon information disclosure.

Although the research perspective of this paper is relatively new and the theoretical basis is relatively solid, there are some limitations. Firstly, this paper examines the impact of government regulatory heterogeneity and spatial distance on carbon information disclosure in the Chinese heavy pollution industry, respectively. However, whether this kind of influence also exists in China’s light pollution industry or non-pollution industry cannot be judged, which may be a limitation of this study. This question needs to be solved by future research. Secondly, whether spatial distance can regulate the heterogeneity of government regulation remains to be investigated. This may be conducive to the government departments to take more effective and targeted regulatory strategies. Thirdly, although the research data in this paper is enough to support the research conclusion, the latest data can be added to further enhance the representativeness of the conclusion in the future research. 

## Figures and Tables

**Table 1:** Descriptive statistics of variables.

Variables	N	Min	Max	Mean	Standard Deviation
CID	768	0.000	1.000	0.305	0.193
CIDNF	768	0.000	1.000	0.379	0.219
CIDF	768	0.000	1.000	0.178	0.208
GOV	768	8.300	85.300	47.841	17.029
Space	768	0.588	8.063	3.575	1.874
ROA	768	−0.326	0.477	0.048	0.056
LEV	768	0.016	1.112	0.531	0.201
Growth	768	−0.703	1.390	0.123	0.244
Size	768	19.304	28.509	23.447	1.659
First	768	0.050	0.864	0.440	0.165
Bsize	768	1.386	3.526	2.533	0.312
BM	768	−0.067	4.921	0.525	0.395
Beta	768	0.053	1.893	1.100	0.292
MI	768	2.530	9.950	7.055	1.764
Year	768	1.000	30.000	14.938	4.512

Note: CID: carbon information disclosure, CIDNF: non-financial carbon information disclosure, CIDF: financial carbon information disclosure, GOV: government regulation variable, Space: the shortest distance between listed enterprises and the government, ROA: profitability, LEV: financial leverage, Growth: enterprise growth, Size: enterprise scale, First: the proportion of first shareholders, Bsize: board scale, BM: book-to-market ratio, MI: market process MI, Year: year listed.

**Table 2:** Correlation analysis results of variables.

Variables	CID	GOV	Space	ROA	LEV	Growth	Size	First	Bsize	BM	Beta	MI	Year
CID	1												
GOV	0.001	1											
Space	0.099 ***	−0.203 ***	1										
ROA	−0.104 **	0.090 **	−0.058	1									
LEV	0.104 ***	−0.054	0.166 ***	−0.549 ***	1								
Growth	−0.009	0.008	−0.035	0.262 ***	0.008	1							
Size	0.354 ***	0.036	0.117 ***	−0.160 ***	0.457 ***	−0.032	1						
First	0.229 ***	0.008	0.061 *	0.029	0.010	−0.093 ***	0.435 ***	1					
Bsize	0.203 ***	−0.162 ***	0.180 ***	−0.148 ***	0.165 ***	−0.057	0.309 ***	0.078 **	1				
BM	0.128 ***	−0.057	0.154 ***	−0.229	0.199 ***	−0.152 ***	0.441 ***	0.097 ***	0.089 **	1			
Beta	0.040	−0.170 ***	−0.021	−0.114 ***	0.000	0.004	−0.195 ***	−0.098 ***	−0.021	−0.134 ***	1		
MI	−0.075 **	0.657 ***	−0.156 ***	0.145 ***	−0.126 ***	−0.040	0.025 ***	0.038	−0.218 ***	−0.016	−0.260 ***	1	
Year	−0.111 ***	0.084 **	0.018	−0.036	−0.032	−0.102 ***	−0.145 ***	−0.284 ***	0.064 *	0.102 ***	−0.125 ***	0.160 ***	1

Note: *** indicates at 1% level; ** indicates at 5% level; * indicates at 10% level (the same below).

**Table 3:** Regression results of overall samples.

Variables	CID	CIDNF	CIDF
GOV	0.094 ** (2.114)	0.091 ** (2.009)	0.072 (1.575)
Space	0.069 ** (1.976)	0.045 (1.263)	0.082 ** (2.264)
ROA	−0.121 *** (−2.773)	−0.115 *** (−2.593)	−0.095 ** (−2.122)
LEV	−0.160 *** (−3.384)	−0.151 *** (−3.150)	−0.127 *** (−2.599)
Growth	0.037 (1.043)	0.031 (0.871)	0.034 (0.929)
Size	0.372 *** (7.294)	0.321 *** (6.216)	0.334 *** (6.348)
First	0.072 * (1.815)	0.106 *** (2.641)	0.006 (0.156)
Bsize	0.082 ** (2.224)	0.087 ** (2.306)	0.053 (1.395)
BM	−0.032 (−0.811)	−0.018 (−0.453)	−0.041 (−1.014)
Beta	0.092 *** (2.598)	0.099 *** (2.741)	0.058 (1.574)
MI	−0.093 ** (−2.012)	−0.068 (−1.455)	−0.100 ** (−2.094)
Year	−0.027 (−0.753)	−0.001 (−0.021)	−0.055 (−1.463)
Constant	−0.792 *** (−6.416)	−0.813 *** (−5.531)	−0.829 *** (−5.496)
Adjusted R Square	0.164	0.139	0.111
F−statistic	13.535 ***	11.340 ***	3.943 ***

**Table 4:** Regression results of public-owned enterprises sample.

Variables	CID	CIDNF	CIDF
GOV	0.112 ** (2.070)	0.099 * (1.810)	0.096 * (1.744)
Space	0.022 (0.530)	−0.010 (−0.249)	0.058 (1.353)
ROA	−0.083 (−1.497)	−0.086 (−1.549)	−0.054 (−0.957)
LEV	−0.075 (−1.277)	−0.061 (−1.025)	−0.072 (−1.192)
Growth	0.040 (0.936)	0.033 (0.760)	0.038 (0.863)
Size	0.338 *** (5.614)	0.280 *** (4.594)	0.317 *** (5.132)
First	0.061 * (1.202)	0.099 * (1.934)	−0.006 (−0.108)
Bsize	0.031 (0.722)	0.054 (1.235)	−0.007 (−0.158)
BM	0.041 (0.822)	0.035 (0.701)	0.036 (0.715)
Beta	0.090 ** (2.039)	0.084 * (1.884)	0.072 (1.590)
MI	−0.100 * (−1.758)	−0.056 (−0.976)	−0.128 ** (−2.193)
Year	0.013 (0.304)	0.022 (0.492)	−0.001 (−0.032)
Constant	−0.725 *** (−4.508)	−0.692 *** (−3.781)	−0.769 *** (−4.003)
Adjusted R Square	0.131	0.110	0.088
F-statistic	7.904 ***	6.660 ***	5.436 ***

**Table 5:** Regression results of non-public-owned enterprises sample.

Variables	CID	CIDNF	CIDF
GOV	0.042 (0.502)	0.074 (0.874)	−0.018 (−0.206)
Space	0.191 ** (2.428)	0.202 ** (2.525)	0.110 (1.348)
ROA	−0.074 (−0.887)	−0.058 (−0.688)	−0.071 ** (−0.814)
LEV	−0.392 *** (−4.503)	−0.352 *** (−4.003)	−0.311 *** (−3.439)
Growth	0.001 (0.013)	0.009 (0.136)	−0.011 (−0.161)
Size	0.253 *** (2.637)	0.225 ** (2.311)	0.205 ** (2.060)
First	0.062 (0.832)	0.069 *** (0.915)	0.031 (0.395)
Bsize	0.140 * (1.790)	0.056 (0.705)	0.209 ** (2.587)
BM	−0.148 * (−1.739)	−0.133 (−1.539)	−0.118 (−1.338)
Beta	0.196 *** (2.791)	0.254 *** (3.5791)	0.045 (0.619)
MI	0.009 ** (0.104)	−0.047 (−0.543)	0.084 (0.954)
Year	−0.090 (−1.191)	−0.013 (−0.165)	−0.203 ** (−2.594)
Constant	−0.711 *** (−2.945)	−0.725 ** (−2.479)	−0.691 ** (−2.439)
Adjusted R Square	0.170	0.149	0.105
F−statistic	4.658 ***	4.130 ***	3.093 ***

**Table 6:** Sensitivity test results.

Variables	Overall Sample	Public-Owned Enterprise Sample	Non-Public-Owned Enterprise Sample
GOV	0.001 * (1.908)	0.002 ** (2.166)	0.001 (0.187)
Spacer	0.001 * (1.936)	0.001 (1.158)	0.023 ** (2.191)
ROA	−0.291 * (−1.746)	0.113 (0.534)	−0.206 (−0.840)
LEV	−0.084 * (−1.761)	−0.048 (−0.782)	−0.359 *** (−4.586)
Growth	0.044 (1.479)	−0.012 (−0.349)	−0.005 (−0.108)
Size	0.013 ** (2.087)	−0.007 (−0.888)	0.042 *** (3.019)
First	−0.075 (−1.532)	0.002 (0.030)	0.013 (0.165)
Bsize	0.117 *** (5.312)	0.013 (−0.451)	0.031 (0.668)
BM	−0.005 (−0.230)	0.021 (0.691)	−0.053 ** (−2.004)
Beta	−0.015 (−0.600)	0.013 (0.460)	0.135 *** (2.910)
MI	−0.011 * (−1.870)	−0.011 (−1.496)	0.004 (0.387)
Year	−0.001 (−0.203)	0.003 (1.547)	−0.006 * (−1.959)
Constant	−0.165 (−1.137)	0.399 ** (2.333)	−0.768 *** (−2.868)
Adjusted R Square	0.053	0.044	0.192
F−statistic	3.541 ***	3.319 ***	3.997 ***
